# Dinuclear complex-induced DNA melting

**DOI:** 10.1186/s12951-023-01784-8

**Published:** 2023-01-23

**Authors:** Niklas Biere, Dennis Kreft, Volker Walhorn, Sabrina Schwarzbich, Thorsten Glaser, Dario Anselmetti

**Affiliations:** 1 Experimental Biophysics & Applied Nanoscience, Faculty of Physics, Bielefeld University, 33615 Bielefeld, Germany; 2 Lehrstuhl für Anorganische Chemie I, Faculty of Chemistry, Bielefeld University, 33615 Bielefeld, Germany

**Keywords:** DNA, AFM, Molecular recognition, Electrospray ionization, Biomolecules

## Abstract

Dinuclear copper complexes have been designed for molecular recognition in order to selectively bind to two neighboring phosphate moieties in the backbone of double strand DNA. Associated biophysical, biochemical and cytotoxic effects on DNA were investigated in previous works, where atomic force microscopy (AFM) in ambient conditions turned out to be a particular valuable asset, since the complexes influence the macromechanical properties and configurations of the strands. To investigate and scrutinize these effects in more depth from a structural point of view, cutting-edge preparation methods and scanning force microscopy under ultra-high vacuum (UHV) conditions were employed to yield submolecular resolution images. DNA strand mechanics and interactions could be resolved on the single base pair level, including the amplified formation of melting bubbles. Even the interaction of singular complex molecules could be observed. To better assess the results, the appearance of treated DNA is also compared to the behavior of untreated DNA in UHV on different substrates. Finally, we present data from a statistical simulation reasoning about the nanomechanics of strand dissociation. This sort of quantitative experimental insights paralleled by statistical simulations impressively shade light on the rationale for strand dissociations of this novel DNA interaction process, that is an important nanomechanistic key and novel approach for the development of new chemotherapeutic agents.

**Supplementary Information:**

The online version contains supplementary material available at 10.1186/s12951-023-01784-8.

## Introduction

The design of new cytostatic compounds with novel mechanisms of action for treatment of cancer has been an ongoing task, in which maximum apoptosis effectiveness for various tumor types is desired, as well as to limit the damage to the surrounding tissue [1–3]. Therefore, a new class of molecules had been synthesized, imitating the hydrolytic cleaving mechanism of metalloenzymes to the backbone of DNA, but regulated by the steric structure of the complex and even tunable by the choice of metal cores and terminal donors [4–7]. This way, cytotoxic effects by blocking DNA polymerase could be anticipated [8].

The complexes have been synthesized by coordination chemistry with metal ions such as Ni^II^ [5, 8], albeit in the following investigations, complexes with Cu^II^ were chosen, since their impact on DNA was shown to be less radical and more feasible to observe [4]. Metal ions display differing hydrolytic cleaving reactivities and can therefore be used to tune the effectiveness to the targeted cancer type [9–11].

The interaction of these complexes with dsDNA has been investigated in depth employing various methods, although exact details of the binding modalities could not finally be uncovered. Therefore, this report aims at employing AFM under UHV conditions to ultimately shed light on the binding scheme, with its unmatched high-resolution capabilities.

In the previous work of Jany et al. [4], investigations using techniques including polymerase chain reaction (PCR), gel electrophoresis, cell arrays and even optical and magnetic tweezers showed that the complexes indeed bind successfully to DNA and cause cell toxicity. The binding was shown to be irreversible and to cause the strands to form intra- and interstrand aggregation.

Since the detailed structural peculiarities of DNA strands are far below the resolution limit of conventional microscopy, AFM is the method of choice for the structural elucidation without further treatments. To get access to details below the grooves, one needs to get rid of adverse influences of ambient conditions, i.e., moisture adsorbate layers, which is only provided by environments of liquids or vacuum. Indeed, measurements under liquid conditions showed remarkable detailed images [12, 13]. There have also been a few investigations of DNA under vacuum [14], though these were mostly limited to the use of substrates that provide strong adhesion of biomolecules, like mica [15–17], since the focus mostly lays on the properties of the strands under physiological conditions before adsorption. Also, the sample preparations relied on the coating and drying of solutions still under ambient conditions, since DNA is not suited for thermal evaporation.

In surface science, a relatively new method of UHV in-situ sample preparation employs the principles of electrospray ionization, originating from mass spectrometry. Hereby, a solution of the desired molecules gets inserted into vacuum where droplet Coulomb fission is provoked by high electric fields [18]. Ideally, the entire solvent evaporates before the molecules reach the sample, where they adhere gently to the clean surface, without contaminants of residual solvent [19–22]. With this new and innovative sample deposition method available, DNA can finally be investigated with extraordinary high resolution [23].

## Results

**Fig. 1:**
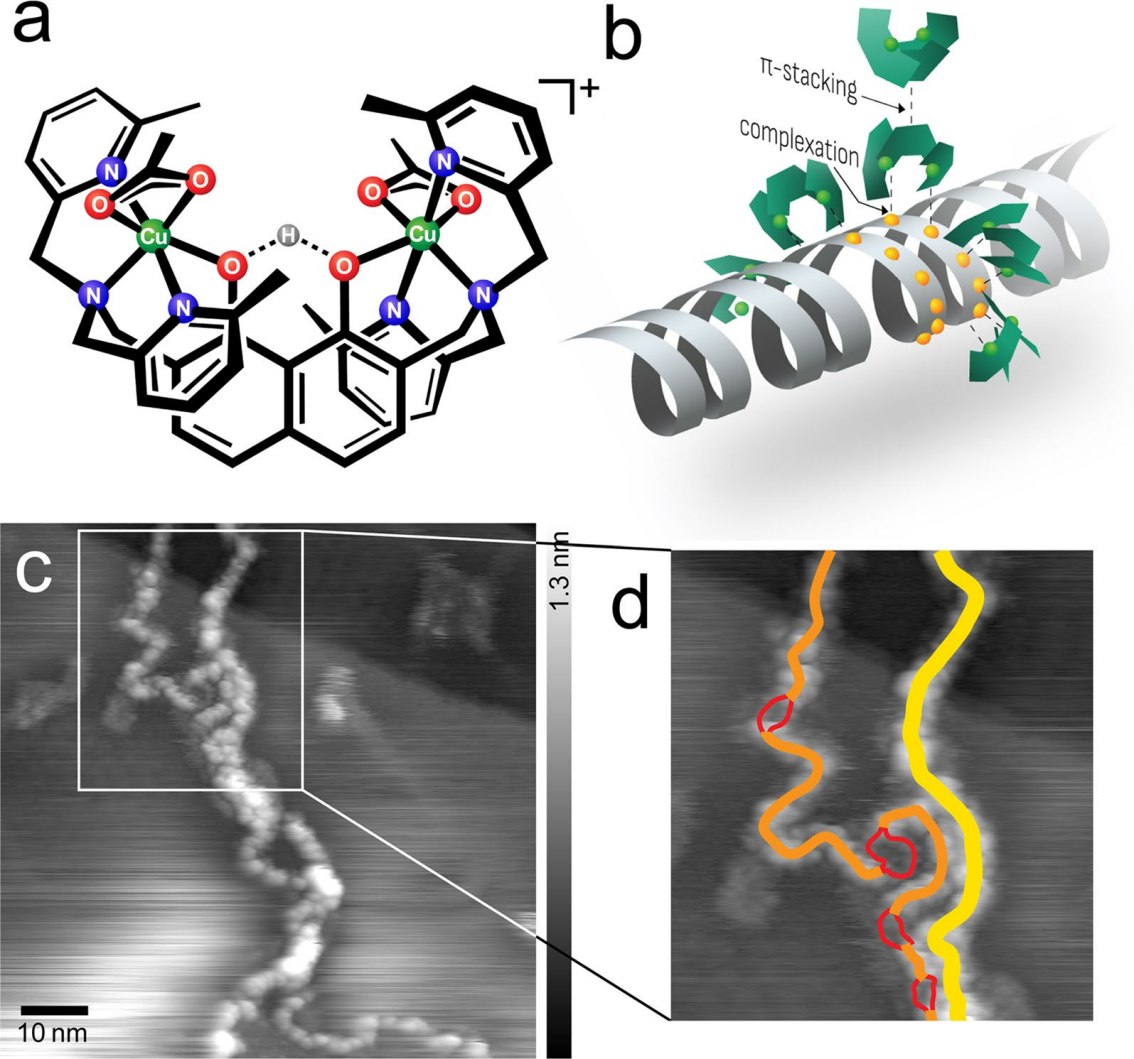
**a** Chemical structure of the [(Htom^Me^){Cu(OAc)}_2_]^+^ complexes. The descriptor for the complexes changes according to the chemical surroundings, for clarification see Additional file 1: Figure S1. **b** Model of binding modes of the complex molecules to adsorb to DNA and to each other. **c** UHV-AFM topographic image of the agglomeration of multiple strands (frequency shift setpoint df =− 2.5 Hz, oscillation amplitude A = 14.1 nm, resonance frequency f_0_ = 259.0 Hz) and the dedicated interpretation **d**: two double strands (yellow, height 0.6 nm), one singular ds (orange, height 0.4 nm), melting bubble dissociation in two single strands (red, height 0.1 nm)

Figure 1a, b illustrate the design of the [(Htom^Me^){Cu(OAc)}_2_]^+^ complexes, with two metal cores that are positioned by the naphthalene backbone at a distance of 0.6–0.7 nm, providing the precise molecular recognition ability of two neighboring phosphate moieties of the DNA backbone. Each of the two pyridyl groups coordinate around the metal atoms in such a way that they sterically limit the hydrolytic cleavage reaction and hamper the nicking of the strand [24].

A solution of λ-phage DNA solution (Lambda DNA Hind III Digest, Sigma-Aldrich, 12.5 ng/µl) (λ-DNA) was treated with a 0.4 nM solution of the dinuclear copper complexes [(Htom^Me^){Cu(OH_2_)}_2_]^3+^, with an incubation time of 45 h, as this was determined to display an indicated onset of expected effects to the DNA. Regarding the concentration dependent influence of the complexes to DNA, a dilution series can be found in the Additional File 1: (Figure S2).

### Backside bonding

A result that was already expected from investigations under ambient conditions was the interstrand agglomeration [4]. The high-resolution images taken under UHV conditions in Fig. 1c, d show how multiple strands align in order to agglomerate, which can be geometrically traced to individual strands.

As Jany et al. already speculated, this effect results from π-stacking effects of the outwards-oriented naphthalene und pyridyl residue moieties of [(Htom^Me^){Cu(OAc)}_2_]^+^. X-ray diffraction showed that the freely rotatable pyridyl groups orient in such a way that, together with the center naphthalene, they form three parallel ridges, which would allow two molecules to attach by coercing multiple π-stacking interactions.

### Melting bubbles

**Fig. 2:**
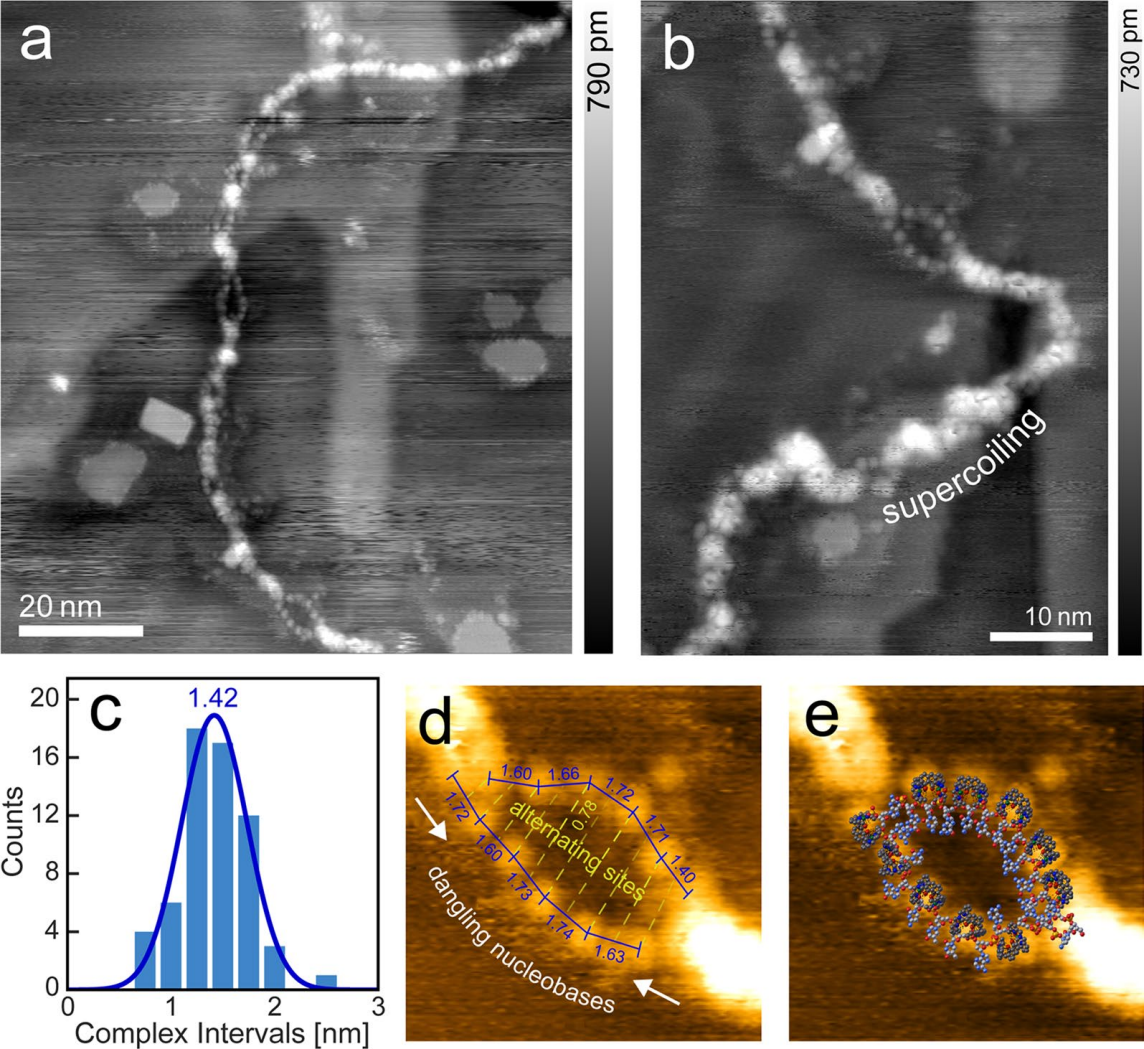
**a** Dissociated melting bubbles along a double strand of λ-DNA (taken under UHV, df =− 2.0 Hz, A = 14.1 nm, f_0_ = 259.0 Hz). **b** Closer look at one of the larger bubbles, including an area of supercoiled buildup. **c** Distribution of interval distances between the protrusion features along the bubbles, with a mean of 1.42 nm. **d** Distances and antiphasic patterning of features. The larger than average distance of 0.78 nm can be attributed to observations where complex coated ssDNA regions appear elongated [25]. **e** Overlay of a possible molecular model to match the observed topography [DNA model from [26] (1BNA)]

Another remarkable feature is introduced in Fig. 2. Along the strand appear several partial fissions of various length into thinner strands, with these sectors ranging in length from 1 to 8 nm. They are not only characterized by a reduced height (0.1–0.2 nm in contrast to 0.4 nm of the main strand), but also by a string of bead-like appearance with equidistant protrusions along a strand.

The phenomenon of sectoral dehybridization of DNA double strands, known as melting-, denaturation- or breathing bubbles, is well-known in DNA science [27]. It is crucial to DNA replication, by introducing insertion point for single strand interacting polymerase enzymes [28, 29] and is often connected to AT-rich (adenine, thymine) DNA regions, which are, due to two instead of three hydrogen bonds, slightly less strongly bound than their GC-counterparts [30] (guanine, cytosine).

They were observed indirectly (by fluorescence quenching [31, 32]) and directly (TEM [25] and AFM [33]), mainly in circular DNA and plasmids, where the openings are induced by twist stress. They appear also in linear DNA [34], caused statistically by molecular motion [27, 35–37].

The height of the measured protrusions lies with 0.2 nm well below the one measured for a double strand in air (1 nm) and vacuum (0.4 nm). In addition, the average interval of the protrusions is with 1.42 nm too large for a single nucleobase length and too low for a double helix pitch, but nicely matches the twofold distance between two neighboring phosphate groups. Therefore, we conclude that the “melted bubble” sections indeed consist of dehybridized double DNA strands into two single strands, where the beads representing the coordinatively bonded [(Htom^Me^)Cu_2_]^3+^ molecules. It shall also be noted that upon closer inspection, a zigzag type arrangement of antiphasic binding sites along the single strands can readily be discerned (see Fig. 2d).

### Reasoning of the melting bubbles

With the dissociated sections making up approximately 12% of the contour length of a strand, which is even above the usual occurrence rate of single strand regions in linear DNA of 4% in supercoiled form [25], the observation of the formation here does not appear to be a purely statistical process.

Melting bubbles do not appear frequently under ambient conditions and are obviously not caused by the UHV conditions, rough handling or freezing as additional measurements have shown. To the best of our knowledge, it has not been shown in literature where DNA adsorbed to low adhesion substrates was subjected to vacuum [38, 39].

Since they do not structurally change over time, it can also be excluded that they are the result of tip-induced damage by the scanning motion. Therefore, a correlation to the presence of the metal-coordinated backbone binding complex molecules is comprehensible.

### DNA behavior in vacuum

To correctly assess these findings, it is essential to take into account the behavior of dsDNA in vacuum environment, as well as the influence of the substrate.

It is widely known that dsDNA in environments of low humidity transitions into a more densely packed formation, the so-called A-form. This is caused by the lower availability of stabilizing water molecules and ions surrounding the negatively charged backbone. A lower helical pitch distance and a more pronounced major groove are characteristic for the A-form [40, 41].

Since it is the most common choice of substrate for investigations under ambient conditions, most AFM-works of DNA in vacuum conditions feature the DNA adsorbed onto functionalized mica. This heavily influences the structural properties of the adsorbed strands, whether it conserves the physiological B-form [15] or changes the elastical properties derived from persistence lengths and equilibrated geometries [42]. The reason is that in order to attach DNA to mica, it is necessary to use a functionalization, to avoid the repelling negative DNA charges of the phosphate backbone as well as of the mica (${\text{SiO}}_{x}^{-}$). Therefore, different methods are employed, that offer different affinities to the DNA. This ranges from less invasive approaches by using divalent cations [12] like Mg^2+^ or Ni^2+^, to the use of strong bonding surface modifications like APTES [42], ODA [34], PLL [43] or PLO [44]. The strength of the bonding influences the observed geometry, since the stronger the bonding, the less dynamics is allowed after adsorption. This leads to 2D equilibrated strands on ionic functionalization and 3D projected strands on covalent bonding surfaces. Electrically neutral substrates like Au or HOPG offer little to no attraction to DNA, disregarding mirror-dipoles and associated van-der-Waals forces, thus, making them mostly unsuitable for use under ambient and liquid conditions. In UHV on the other hand, gold is of great advantage, since it can be ultra-cleanly prepared and atomically controlled in-situ, while mica introduces a distinct roughness that is highly disadvantageous to high-resolution measurements.

Since literature is sparse, regarding the interaction of DNA with gold, its behavior during electrospray ionization and even its behavior in vacuum conditions, an appropriate series of tests of deposition on different substrates has been conducted.

### Gold

**Fig. 3:**
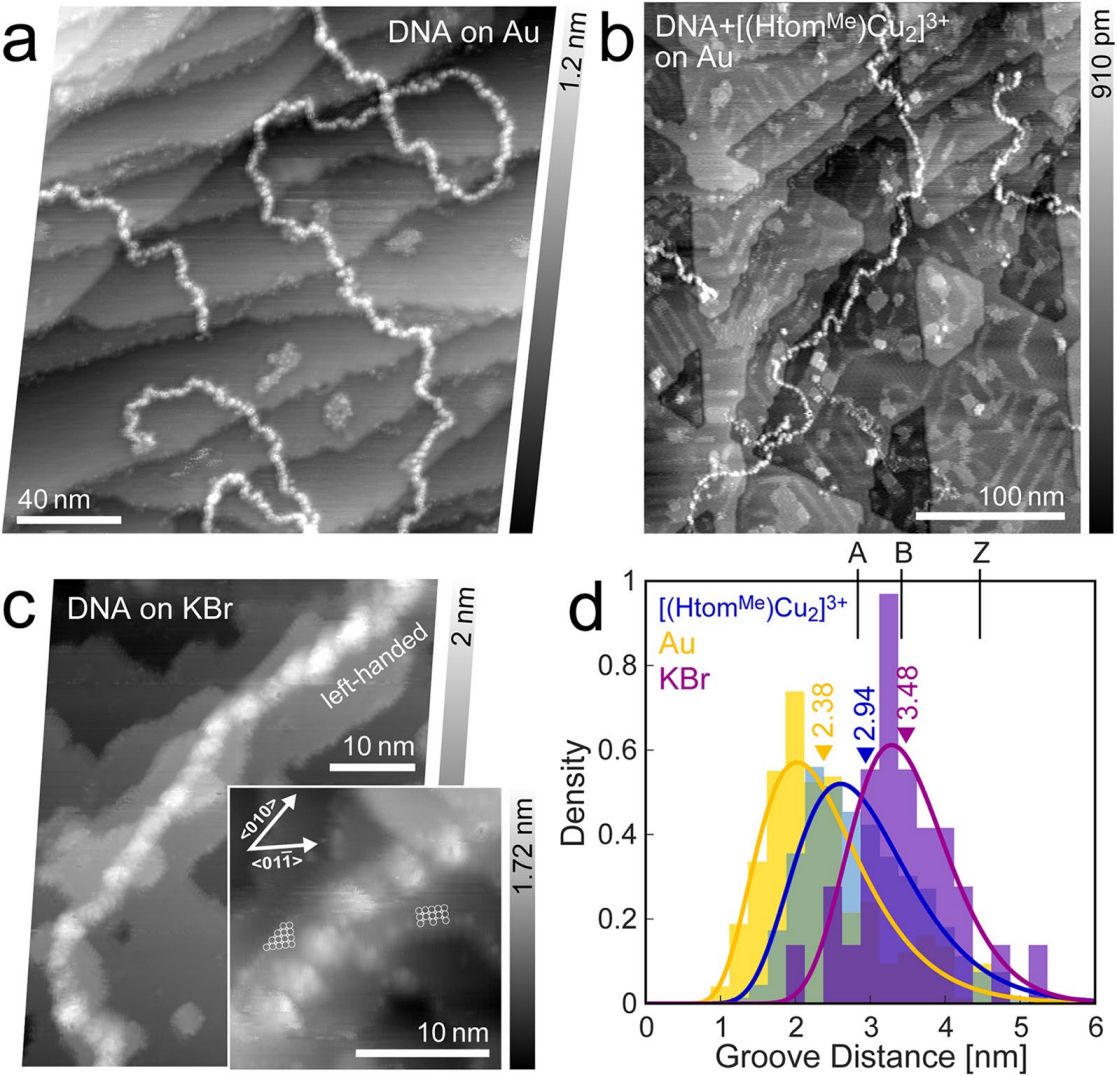
λ-DNA subjected to UHV conditions on different substrates. **a** Untreated DNA on Au(111) (df =− 6.2 Hz, A = 12.6 nm, f_0_ = 286.3 Hz). **b** [(Htom^Me^)Cu_2_]^3+^-treated DNA on Au(111) (df =− 2.0 Hz, A = 14.1 nm, f_0_ = 259.0 Hz). The residues on the otherwise flat atomic planes are likely due to residual complex molecules. **c** DNA on potassium bromide (df =− 8.6 Hz, A = 4.7 nm, f_0_ = 302.0 Hz, inset: df =− 20.2 Hz, A = 1.9 nm, f_0_ = 302.0 Hz). **d** Distribution of groove distances, along with corresponding mean values for different substrates (Au, KBr)

A string of bead-like appearance characterizes the pristine DNA-strands on gold (Fig. 3a). The grooves between the features correlate to the major groove [38] while smaller features could not be resolved, due to instability of the sample. Another explanation features for the beads to be correlated to electrostatic forces from residual ions, which are known to reside primarily in the grooves [45, 46]. Caused by the low interaction between strand and substrate, the strands are easily moved during scanning, which prevents higher resolution. Figure 3d shows a distribution of measured groove distances. The mean value of 2.38 nm is in agreement to the expected pitch value range for A-form transformed DNA (2.82 nm [47]). The associated strand height measures to about 0.5 nm.

### Potassium bromide

As a reference, pristine λ-DNA strands were additionally deposited on an in-situ cleaved surface of KBr. Strands can clearly be identified, with features that resemble the major groove, even despite signs of minor rearrangement of ion plateaus, possibly caused by residual solvent hitting the surface (Fig. 3c).

A statistical assessment of groove distances shows high agreement to the expected full turn distance of 3.4 nm of the B-form DNA-configuration.

For most parts, the right-handedness can be readily observed. However, upon closer look, segments of differing groove distance and even left-handedness, resembling A- and even the Z-form of dsDNA [41], can be additionally discerned. This is known to occur under high salt concentrations at GC-rich sections [48] and even on Ni-functionalized mica [49].

The expected vacuum-induced transition to the A-form is apparently hampered for the most part, due to an abundance of ions that act stabilizing to the physiological B-form and being able to retain a commensurate number of water molecules from evaporating [45].

The measured dsDNA height is about 0.6 nm, due to flattening from adhesion forces and embedding into residual solvent.

### *[(Htom*^*Me*^*)Cu*_*2*_*]*^*3*+^*-bound DNA*

Aside from the already discussed strand aggregations and bubble formations, the DNA bonded with [(Htom^Me^)Cu_2_]^3+^ exhibits a similar string of bead-like appearance, away from the larger aggregations (Fig. 3b). A statistical analysis of their groove distances leads to a mean value of 2.94 nm. While the pitch can slightly be influenced by the base sequence [48], this is far in between the untreated DNA on gold that has taken on the A-form and the presumably conserved B-form on KBr.

There were no differences in persistence lengths noticeable for each of the three cases, residing each time around 10 nm.

## Discussion

### Freezing of B-conformation

As stated above, the complexes are designed in such way that they recognize and selectively bind to two consecutive phosphate moieties of the backbone of a DNA strand. For such a specific level of molecular recognition to a specific binding site geometry, not only the distance of the metal atoms is important, but also their metal-coordination site angles. Since the acetate groups, which are present in the unsolvated state, point outward at 110° (see Additional file 1: Figure S4), the binding could favor sites larger than the copper distance.

The measurements of untreated DNA showed that DNA tends to transition into a different configuration when not subjected to stabilizing factors. One of these stabilizing factors was shown to be salt ions, while they may not work directly but rather by binding residual water molecules necessary to stabilize the B-form. Here, the complexes appear to act in a similar fashion to the backbone of the DNA. Upon conformational change, a smaller phosphate distance would be highly unfavorable for the binding kinetics. Since the binding already occurred during the incubation in liquid environment and was shown to be irreversible [4], the adsorbed complexes act like a brace, forcing the phosphates to the former larger distance, and therefore partially conserving the B-conformation, at least to some degree, an effect well described for other compounds [50]. Incidentally, this effect might also be related to the high occurrence of melting bubbles.

### Melting bubbles

The reason for the B-to-A conformational change can be found in the puckering of the furanose rings of the backbone. In the hydrated form they take on a C2’-endo form, whether without the influence of water molecules, the energetically favorable conformation is the C3’-endo form, which is characterized by a switched angle between the 2’- and 3’-carbon atoms (illustrated in Additional file 1: Figure S5). This altered angle leads to a reduced geometrical distance between the phosphates from 6.72 [26] to 5.85 Å [51], thus the denser packing in A-configuration [47, 52, 53].

Without the stabilizing influences of water molecules, the former state is now energetically unfavorable, but nonetheless enforced by the complexes. This way, an angular mechanical strain is introduced onto the base pairs, which are prone to dissociating due to rotational motions [54]. We argue that it is actually favored for specific segments to break up and melt into single strands in order to relief this mechanical stress.

Since there are hints that residual solvent hits the surface before evaporating completely, the molecules are already in contact with the substrate when complete evaporation occurs. For a transformation to happen now, it would require extensive on-surface dynamics, which the employed gold substrate indeed allows for. When bubbles open up, the overall twist sum of helical turns needs to be conserved. Still, after adsorption, the strands are somewhat restrained in their freedom of motion, despite the low adhesive force of the substrate. This would cause a local supercoiling buildup, which can indeed be observed locally (Fig. 2b).

**Fig. 4:**
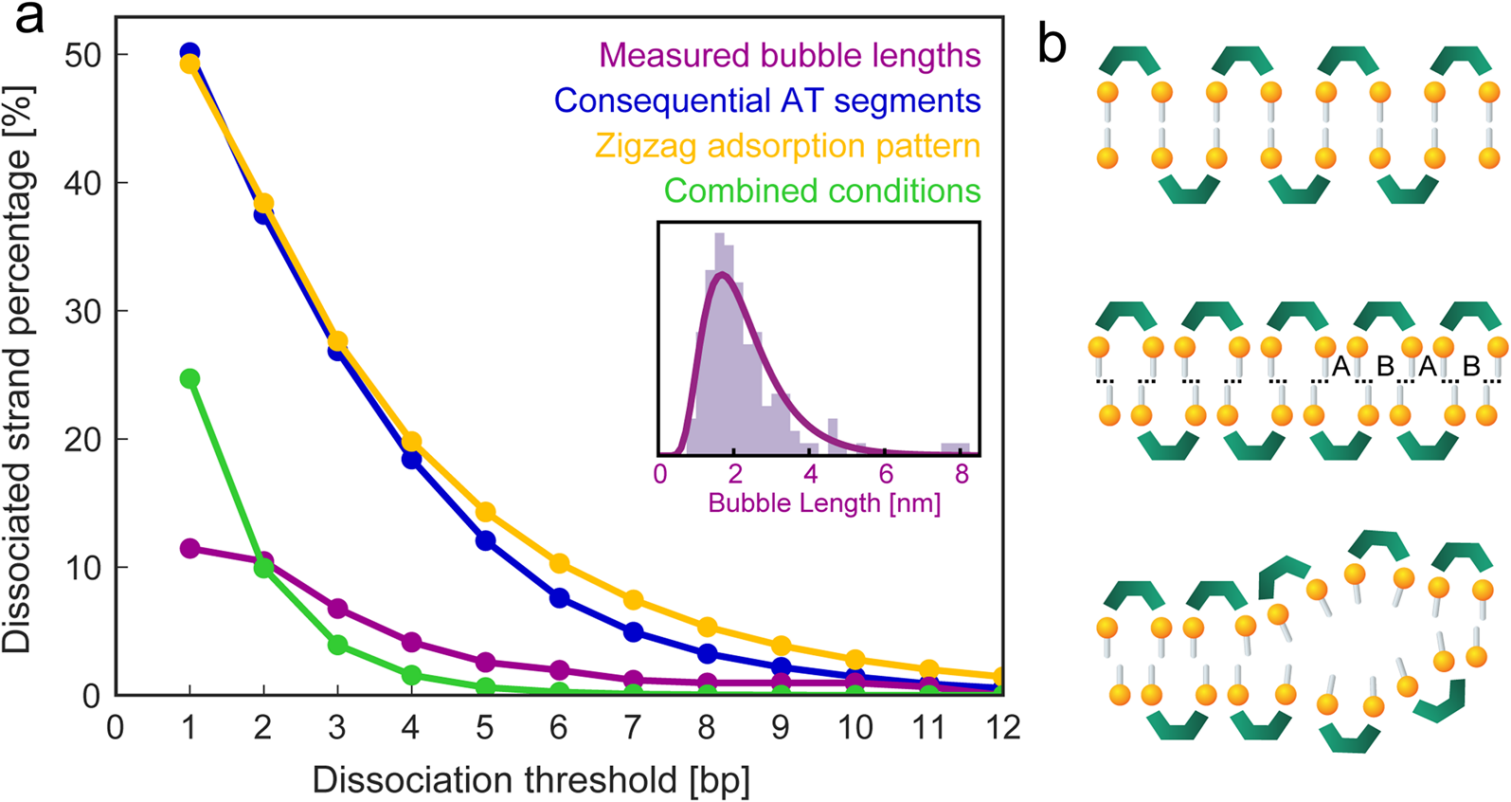
**a** Contour length percentage along a strand when summing up dissociated bubble segments from a certain threshold value (purple). Segments with consequential AT base paring along the λ-phage genome sequence (blue). The percentage of segments with odd complex adsorption pattern, derived from a statistical simulation (yellow). Percentage of areas where both conditions are met at the same time (green), fitting well to the actual observed bubble lengths. The dip at 1 bp accounts for the visual counting threshold. Inset: Distribution of melting bubble lengths. The relation between base pair and nm of 1 bp $\widehat{=}$ 0.71 nm was determined from the interval distance in Fig. 2c. **b** Explanation model for the bubble formation. Top: Zigzag adsorption pattern of complex molecules. Middle: Reduced phosphate distance by partial A-to-B transformation introduces tensile stress on the base pairing. Bottom: Dehybridization of weaker bound AT-base pairs

To determine possible causes, a statistical analysis correlated the dissociation rate with the occurrence of specific conditions and geometric adsorption patterns. It showed that the percentage of passages of more than two subsequent AT-pairs in the λ-phage genome is 37.5% (Fig. 4a). Two base pairs corresponding here to the smallest observed gap distances, so the phenomenon cannot purely be related to the AT-pair occurrence.

In contrast, with a purely statistical adsorption model, the occurrence of passages of more than two adversely packed complex molecules (equaling three base pairs) is roughly at 38.4%, which is far higher than the observed dissociated areas. When combining these two factors, simulating the adsorption of complex molecules onto the λ-phage genome, the percentage where both of these conditions occur at the same time is about 10%. This fits well with the 12% observed dissociation contour length.

This lets us conclude that an interplay of adsorption pattern and the base pair sequence is responsible for the observed formation of melting bubbles. Having both conditions occur simultaneously makes a strand segment energetically prone for dissociation, with tensile stress on the base pairs that would otherwise not be present when the adsorption pattern was parallel or random, as illustrated in Fig. 4b. Although this statistical method only gives a rough estimation, since it leaves out more complex effects like anisotropic stacking interaction [55] and cooperative area opening [37], it nicely reflects the overall findings.

## Conclusion

Double stranded λ-phage DNA was successfully treated with dinuclear [(Htom^Me^)Cu_2_]^3+^ complexes upon selective metal coordination and deposited in UHV by electrospray deposition onto in-situ prepared Au(111) and investigated by AFM. Binding to the phosphate backbone could be confirmed, as well as the effect of backside π-stacking mediated strand agglomeration.

Interestingly, high amounts of melting bubbles were evidenced, where even individual complex molecules could be identified. The unfolding confirms the expected bonding scheme in contrast to other feasible bonding modes such a quasi-minor groove strand bridging. Aside from coverage of every two phosphates, the binding can take on even the closest expected packing, without any non-cooperative dynamics.

Compared to our previous series of experiments, in which we characterized binding phenomena of dinuclear complexes under ambient conditions, the UHV data presented here exhibit a resolution down to the single base pair level. The observed melting bubbles were explained by the bound complexes blocking the vacuum-induced B-A-transition by shortening of backbones, which leads to the mechanically preferable melting of AT-rich segments coupled with areas of odd packing, where single strand stability is energetically favored over conservation of the double strand conformation.

These quantitative experimental results, paralleled by statistical simulations impressively shade light on the rationale for strand dissociations of a novel DNA interaction process.

Besides these findings, the presented study demonstrates the value of such an approach of observing the behavior of individual molecules, employing the high-resolution capabilities of AFM, complementing common ensemble-based techniques. Going forward, this methodology can be applied for various chemotherapeutical agents, in order to gain detailed insights into their nanomechanistic properties, assess their effectiveness in blocking DNA-synthesis and potential to cause overall damage. This way aiding not only the development of new medicinal compounds, but also the associated clearance process through the federal drug agencies.

## Methods

### Complex synthesis

The synthesis of the [(Htom^Me^){Cu(OAc)}_2_](OAc) compound is described elsewhere [5]. The complex (M = 1158.22 g/mol) was dissolved in distilled water (MilliQ) at a concentration of 200 µM before this solution was added to the DNA solution. Incubation times were $\sim$ 45 h.

### Atomic force microscopy

The scanning force images were recorded on a RHK UHV 7500 system (RHK Technology, Troy, USA) with R9 controller electronics. Cantilevers used were Tap300Al-G (Budget Sensors, Sofia, Bulgaria) and NCH-PPP (Nanosensors, Neuchâtel, Switzerland), which were sputtered before use by Ar^+^ ions (8 × 10^–7^ mbar) at 680 eV for 90 s. Images were recorded in AFM non-contact imaging mode, at room temperature and at 90 K (cooled by a liquid nitrogen flow cryostat), at a pressure of 5 × 10^–11^ mbar. They were corrected for thermal drift [56], using Gwyddion SPM image software [57]. Persistence lengths were measured with Easyworm [58].

### Sample preparation

The samples were prepared in-situ on a commercial substrate consisting of a 300 nm layer of Au(111) epitaxially grown onto a sheet of mica (Georg Albert PVD, Silz, Germany). They were precleaned in ethanol and water before transferring to UHV. There they were sputtered for 5 min with Ar^+^ ions at 8 × 10^–7^ mbar und subsequently annealed at 523 K for 2 h, for two cycles and checked for cleanliness before deposition of molecules.

### Electrospray deposition

A solution of λ-phage DNA in MilliQ water (12.5 ng/µl, 396 µl) was treated with the complex solution (40 nmol/l, 4 µl) with a resulting complex concentration of 0.4 nmol/l and was diluted with methanol in a 2:1 ratio immediately before ESI deposition [59]. The system used for the procedure was a commercial MolecularSpray (MolecularSpray Ltd.) setup, with three individually pumped chambers of gradually decreasing pressure (1 × 10^− 1^ mbar, 1 × 10^− 2^ mbar, 1 × 10^− 5^ mbar). The solution is carried by a syringe through PEEK tubing to the capillary where it gets nebulized by a voltage of 2 kV and injected into the vacuum chamber. The syringe provided a flux of 10 µl/min, approximately 500 µl of sample solution was injected.

### Simulation

The statistical simulation was written in Matlab 2018b. The λ-phage genome sequence originates from [60]. Pseudocode is attached in Additional file 1.

## Supplementary Information

**Additional file 1: Figure S1.** Various stages of the (Htom^Me^)^-^ complexes. (a) The basic form of the H_2_tom^Me^ ligand. (b) A methoxymethyl(MOM)-based precursor for synthesis of the metal complexes. (c) The cationic form crystallized with an acetate OAc^−^ counter anion. (d) In aqueous solution the acetate groups are replaces by H_2_O. (e) The copper ions coordinate to the phosphates of the DNA backbone. **Figure S2.** λ-DNA subjected to dinuclear copper complexes at various concentrations. Images taken in air in tapping mode. Same length and height scales apply to each image. (a) Untreated λ-DNA (12.5 ng/µl), strand height 0.6 nm. (b) Final complex concentration 0.7 µM, increase of strand height to 1 nm. (c) 1.4 µM, height 0.8 nm. (d) 2 µM, increase of coagulation (indicated by arrows), height 1 nm. (e) 7 µM, height 1 nm. (f) 14 µM, complete coagulation, height 0.4 - 1 nm. (g) 0.4 nM with an incubation time of 45 h. (h) 4 nM. **Figure S3.** Empirical determination of a suitable complex concentrations: measurements of electrospray depositions of complex-treated DNA under UHV. (a) At 2 µM, a massive residual background is present, the strands appear visibly embedded into a layer (df = - 2.2 Hz, A = 36.1 nm, f_0_ = 257.4 Hz). (b) At 0.4 nM, still a slight residual molecular background is visible, the gold surface appears to be covered (df = - 2.5 Hz, A = 14.1 nm, f_0_ = 259.0 Hz). Although technically there should be less complexes than binding sites to the DNA, due to the obvious excess of molecules, it can be assumed that the strands are maximally coated. **Figure S4.** The [(Htom^Me^){Cu(OAc)}_2_]^+^ complex chemical structure (hydrogen left out) from different perspectives, based on data from [1,2]. (a) Angular view, (b) side view, (c) top view. **Figure S5.** The puckering of the furanose ring of the DNA backbone changes in humid environments. The (*)-marked C2’ atom switches from the front into the back of the ring plane. The designations C2’- and C3’-endo refer to the atom which is positioned on the side of the C5’ atom. Structural X-ray diffraction data from [3] (1BNA, B-DNA) and [4] (116D, A-DNA). **Figure S6.** Pseudocode for the analysis of the λ-genome sequence. Firstly, the sequence is reduced for easier counting, then the counting of segments in dependence of the threshold value length occurs. **Figure S7.** Pseudocode for the adsorption simulation and subsequent analysis. The adsorption routine is depicted in black. Two arrays of singular binding spots, representing the two single strands of one double helix, get subsequently and randomly filled up until no free spots remain. Since the molecules require two adjacent spots, full adsorption will leave randomly distributed singular free spots. In yellow and green depicted are the analysis routines, according to their colors in the graph of Figure 4 in the main paper. The adsorbed strand is reduced for easier identification of molecules. Then two counters are iterated over the arrays, each counting one zigzag phase. The exit conditions for the counting of one segment are that both spots of the same index are either empty or both occupied, meaning the zigzag arrangement would be interrupted either way. In addition, when a GC-pair is present (green).

## Data Availability

The datasets used and/or analyzed during the current study are available from the corresponding author on reasonable request.
