# Supplementary material for: Dinuclear complex-induced DNA melting
Source: J Nanobiotechnology. 2023 Jan 23;21:26. doi: 10.1186/s12951-023-01784-8 (PMC9869567; doi:10.1186/s12951-023-01784-8)
Supplement: Supplementary file 1 — Additional file 1: Figure S1. Various stages of the (HtomMe)- complexes. (a) The basic form of the H2tomMe ligand. (b) A methoxymethyl(MOM)-based precursor for synthesis of the metal complexes. (c) The cationic form crystallized with an acetate OAc− counter anion. (d) In aqueous solution the acetate groups are replaces by H2O. (e) The copper ions coordinate to the phosphates of the DNA backbone. Figure S2. λ-DNA subjected to dinuclear copper complexes at various concentrations. Images taken in air in tapping mode. Same length and height scales apply to each image. (a) Untreated λ-DNA (12.5 ng/µl), strand height 0.6 nm. (b) Final complex concentration 0.7 µM, increase of strand height to 1 nm. (c) 1.4 µM, height 0.8 nm. (d) 2 µM, increase of coagulation (indicated by arrows), height 1 nm. (e) 7 µM, height 1 nm. (f) 14 µM, complete coagulation, height 0.4 - 1 nm. (g) 0.4 nM with an incubation time of 45 h. (h) 4 nM. Figure S3. Empirical determination of a suitable complex concentrations: measurements of electrospray depositions of complex-treated DNA under UHV. (a) At 2 µM, a massive residual background is present, the strands appear visibly embedded into a layer (df = - 2.2 Hz, A = 36.1 nm, f0 = 257.4 Hz). (b) At 0.4 nM, still a slight residual molecular background is visible, the gold surface appears to be covered (df = - 2.5 Hz, A = 14.1 nm, f0 = 259.0 Hz). Although technically there should be less complexes than binding sites to the DNA, due to the obvious excess of molecules, it can be assumed that the strands are maximally coated. Figure S4. The [(HtomMe){Cu(OAc)}2]+ complex chemical structure (hydrogen left out) from different perspectives, based on data from [1,2]. (a) Angular view, (b) side view, (c) top view. Figure S5. The puckering of the furanose ring of the DNA backbone changes in humid environments. The (*)-marked C2’ atom switches from the front into the back of the ring plane. The designations C2’- and C3’-endo refer to the atom which is positioned on [file 12951_2023_1784_MOESM1_ESM.docx]

Supporting Information

**Dinuclear Complex-induced DNA Melting**

Niklas Biere,^1^ Dennis Kreft,^1^ Volker Walhorn,^1^ Sabrina Schwarzbich,^2^ Thorsten Glaser,^2^ and Dario Anselmetti^1,*^

^1^ Experimental Biophysics & Applied Nanoscience, Faculty of Physics, Bielefeld University, 33615 Bielefeld, Germany

^2^ Lehrstuhl für Anorganische Chemie I, Faculty of Chemistry, Bielefeld University, 33615 Bielefeld, Germany

Corresponding author:

*nbiere@physik.uni-bielefeld.de

**Synthesis and Bonding Schemes of Dinuclear Copper Complexes**

**
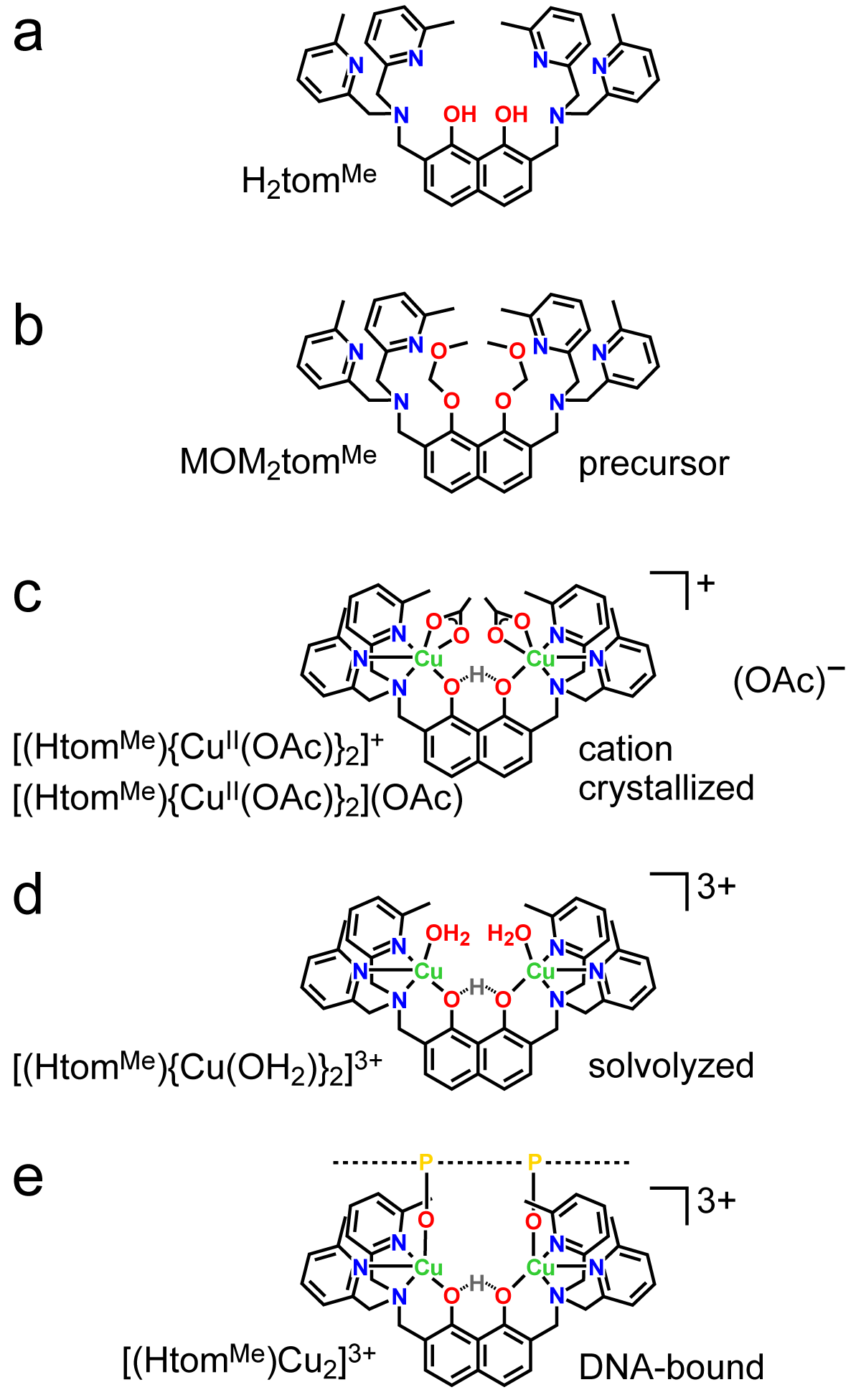
**

***Figure S1.*** *Various stages of the (Htom^Me^)^-^ complexes. (a) The basic form of the H_2_tom^Me^ ligand. (b) A methoxymethyl(MOM)-based precursor for synthesis of the metal complexes. (c) The cationic form crystallized with an acetate OAc^−^ counter anion. (d) In aqueous solution the acetate groups are replaces by H_2_O. (e) The copper ions coordinate to the phosphates of the DNA backbone.*

**Determining the Complex Concentration**

To determine an applicable concentration of complex molecules to DNA, a dilution series on Ni-functionalized Mica were performed in tapping mode under ambient conditions (Figure S2 a-f). With increasing concentration, the visual effect of strand agglomeration strengthened. A final concentration of 2 µM was determined to display a suitable onset of interstrand agglomeration (Figure S2 d, indicated by arrows).

This use of this solution proved to be disadvantageous due to a high background of residual complex molecules. Filtration of the solution lead to obstruction of the capillaries and was therefore not possible to deploy with the electrospray setup. To resolve this issue, the complex concentration was drastically reduced and in turn the incubation time increased from 20 minutes to 45 hours (Figure S2 g-h). A corresponding visual effect was determined at a concentration of 0.4 nM, which was then used for electrospray deposition.


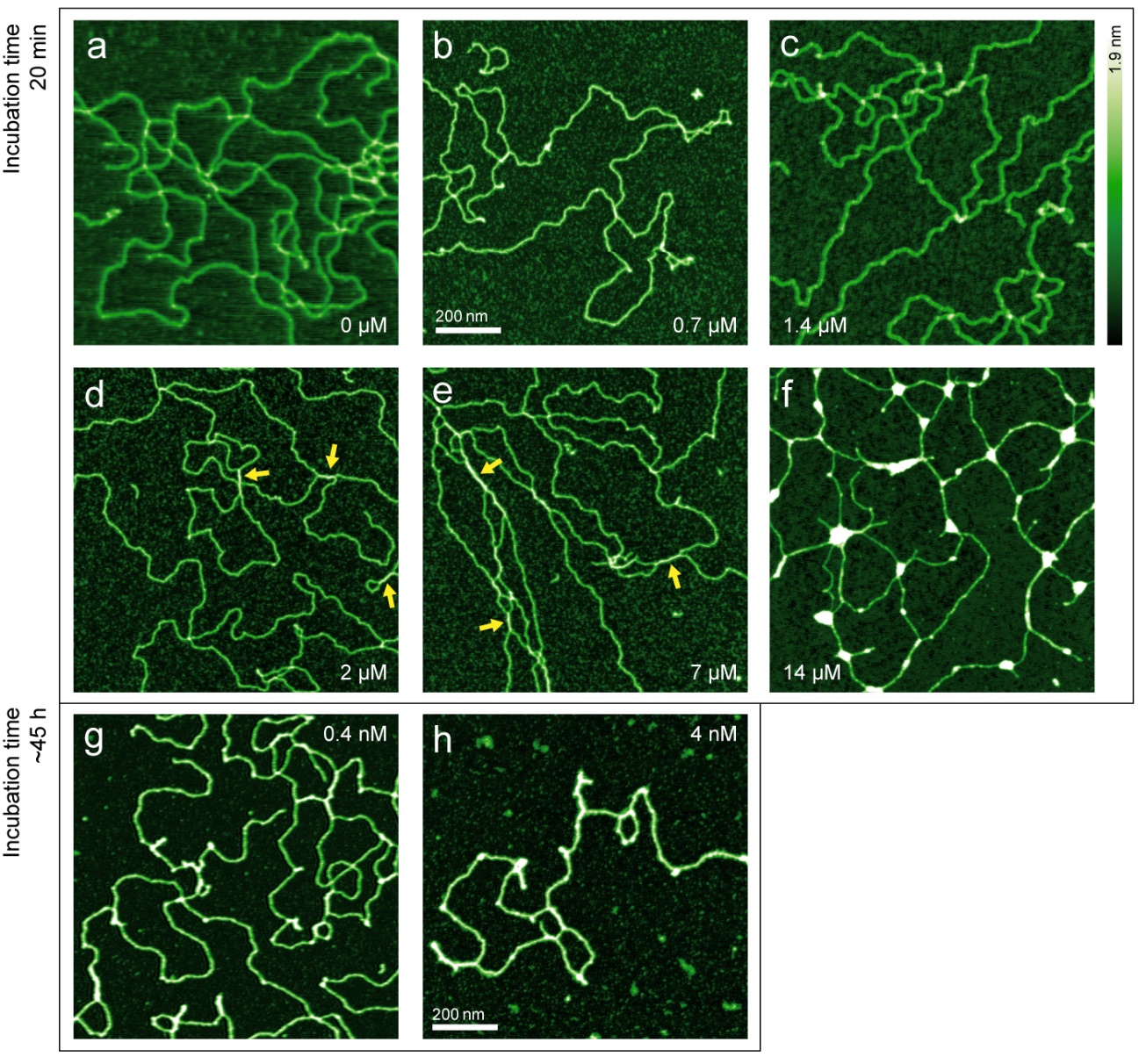


***Figure S2.*** *λ-DNA subjected to dinuclear copper complexes at various concentrations. Images taken in air in tapping mode. Same length and height scales apply to each image. (a) Untreated λ-DNA (12.5 ng/µl), strand height 0.6 nm. (b) Final complex concentration 0.7 µM, increase of strand height to 1 nm. (c) 1.4 µM, height 0.8 nm. (d) 2 µM, increase of coagulation (indicated by arrows), height 1 nm. (e) 7 µM, height 1 nm. (f) 14 µM, complete coagulation, height 0.4 - 1 nm. (g) 0.4 nM with an incubation time of 45 h. (h) 4 nM.*

While 0.4 nM technically yiels a ratio of only one complex to 50.000 binding sites, the considerations of a completely covered strand can still be assumed true: As preliminary measurements have shown a massive excess of molecules at 2 µM (Figure S3). With a calculated ratio of still only 1 complex to 10 binding spots, a large quantity of DNA must be lost during the electrospray deposition process.


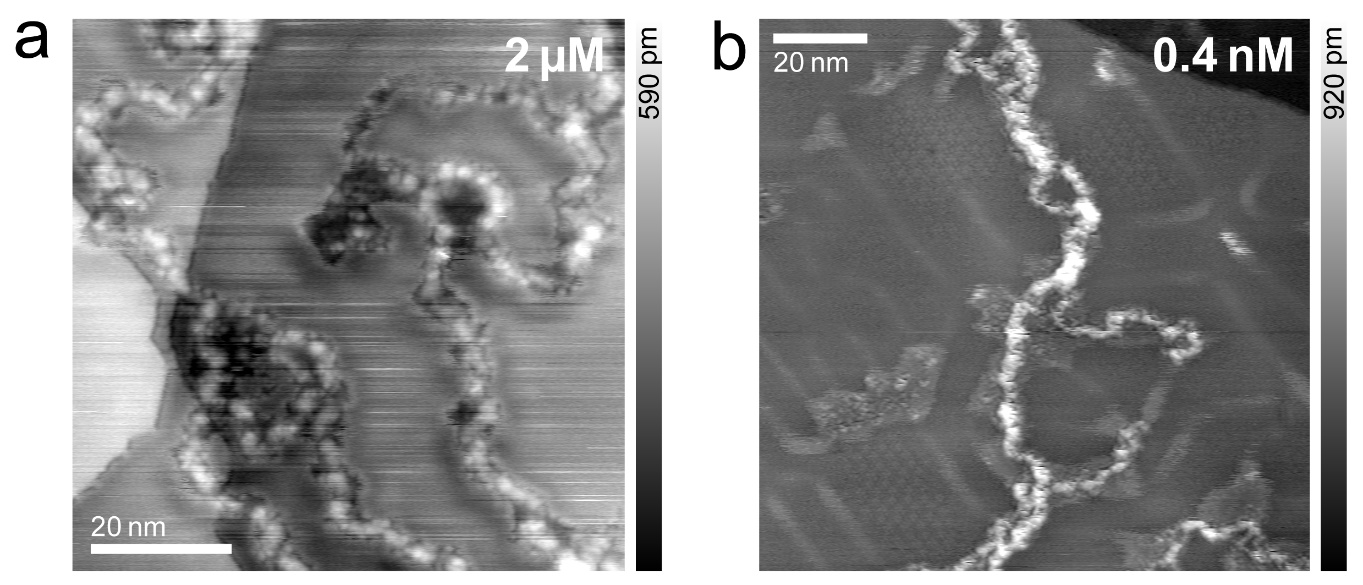


***Figure S3.*** *Empirical determination of a suitable complex concentrations: measurements of electrospray depositions of complex-treated DNA under UHV. (a) At 2 µM, a massive residual background is present, the strands appear visibly embedded into a layer (df = - 2.2 Hz, A = 36.1 nm, f_0_ = 257.4 Hz). (b) At 0.4 nM, still a slight residual molecular background is visible, the gold surface appears to be covered (df = - 2.5 Hz, A = 14.1 nm, f_0_ = 259.0 Hz). Although technically there should be less complexes than binding sites to the DNA, due to the obvious excess of molecules, it can be assumed that the strands are maximally coated.*

**Sterical Structure**

The sterical structure of the complex molecules shows the three aromatic blades that provide π-stacking capability. Images S4 a and b shows the two acetate residues pointing outwards at an angle of 110°, thus making bonding sterically sensitive.


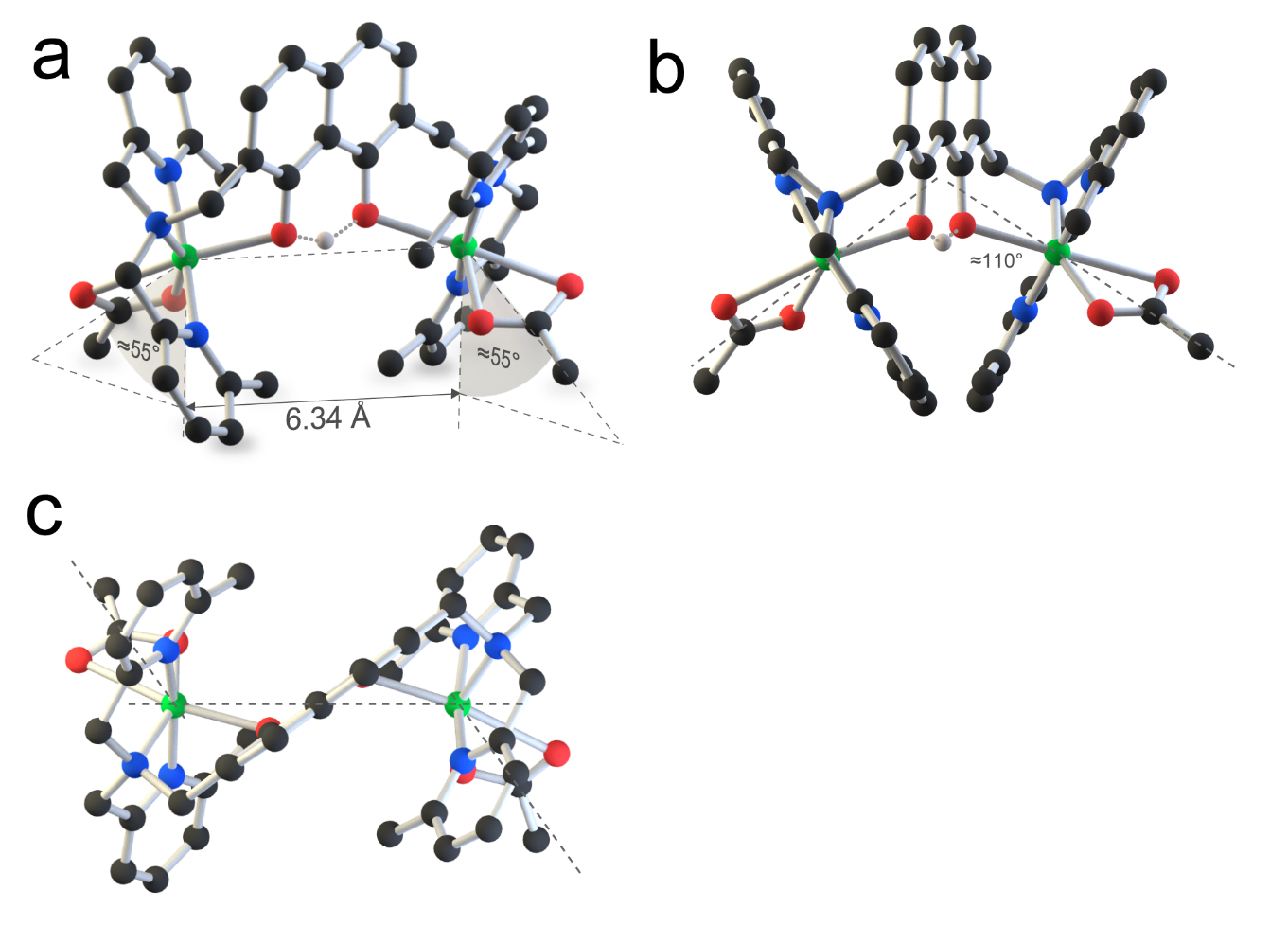


***Figure S4.*** *The [(Htom^Me^){Cu(OAc)}_2_]^+^ complex chemical structure (hydrogen left out) from different perspectives, based on data from [1,2]. (a) Angular view, (b) side view, (c) top view.*

**B-to-A Transition of DNA**

In dry environments, the physiological prevalent B-conformation of DNA transitions into a denser A-conformation. This is characterized by a reduced phosphate distance. The reason herein lies in the puckering of the furanose ring, as shown in Figure S5.

Due to the different puckering, the distance between the phosphorus atoms decreases from 6.72 Å (B-DNA) [3] to 5.84 Å (A-DNA) [4]. The distance of the outward-pointing oxygen atoms changes from 7.31 Å to 6.40 Å accordingly.

**
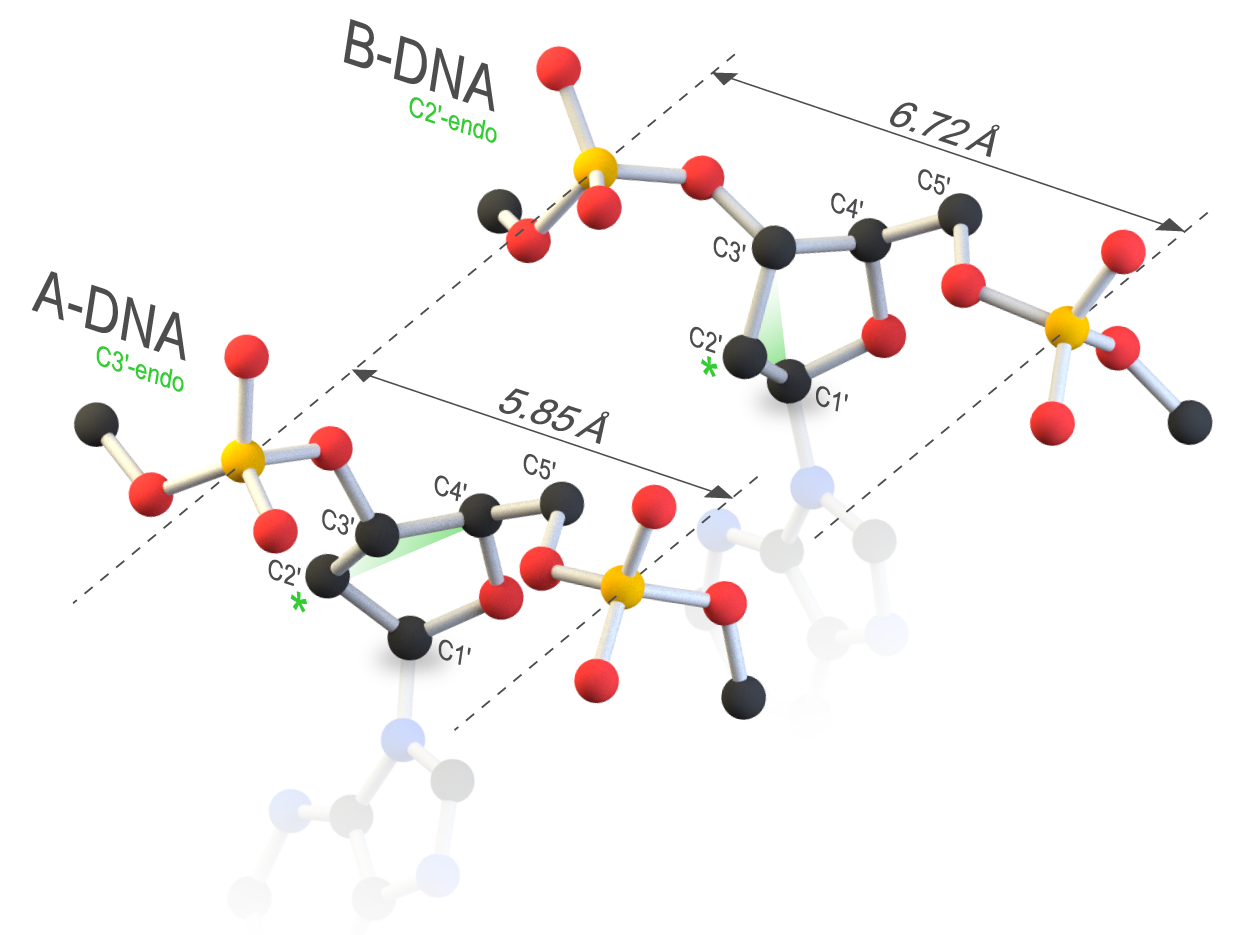
**

***Figure S5.*** *The puckering of the furanose ring of the DNA backbone changes in humid environments. The (*)-marked C2’ atom switches from the front into the back of the ring plane. The designations C2’- and C3’-endo refer to the atom which is positioned on the side of the C5’ atom. Structural X-ray diffraction data from [3] (1BNA, B-DNA) and [4] (116D, A-DNA).*

**Statistical Simulation**

An algorithm simulating a purely statistical irreversible binding of complex molecules to two basepairs until saturation was composed in MATLAB. Each point of the graph in the main paper (Figure 4) shows the percentage of the λ-phage genome that consists of sequences of n consecutive AT- or TA-basepairs or larger (blue). The percentage of a sequence of unweighted binding sites which, after statistical irreversible adsorption of molecules that require two consecutive free binding sites, is occupied by a zigzag type adsorption pattern of n – 1 molecules or larger (where n – 1 molecules occupy n basepairs) is depicted in yellow. The percentage of the sequence where both conditions apply at the same time, meaning an AT-sequence with zigzag type antiphasic packing is denoted in green.

In the following figures, the pseudo algorithms for the statistical analysis and adsorption simulation are explained in more detail.


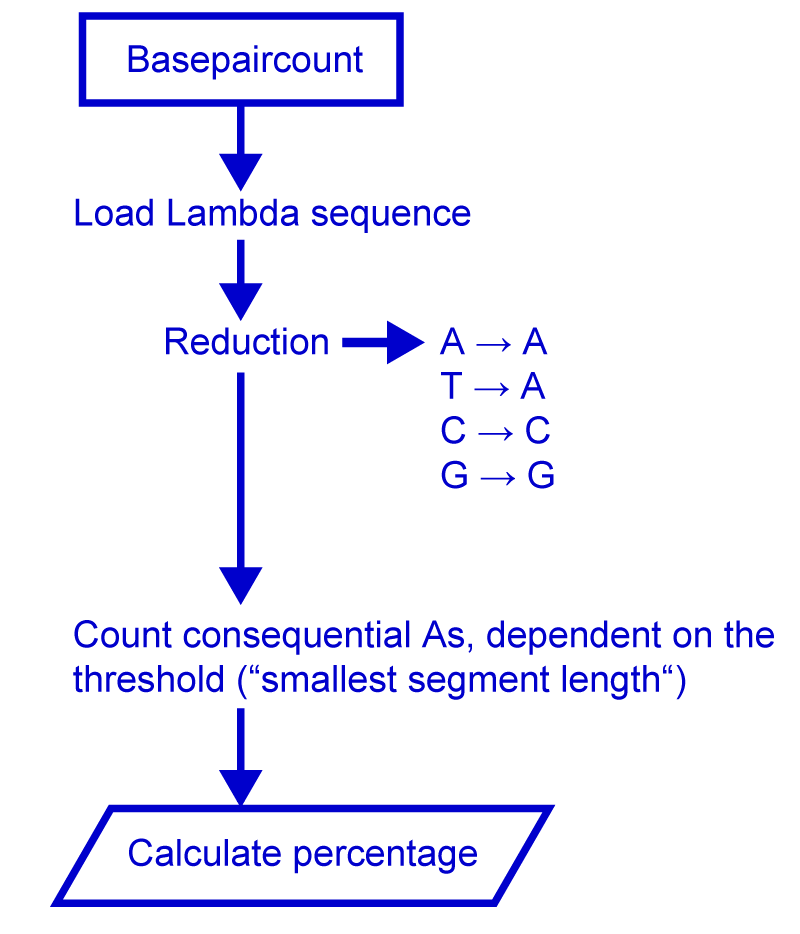


***Figure S6.*** *Pseudocode for the analysis of the λ-genome sequence. Firstly, the sequence is reduced for easier counting, then the counting of segments in dependence of the threshold value length occurs.*


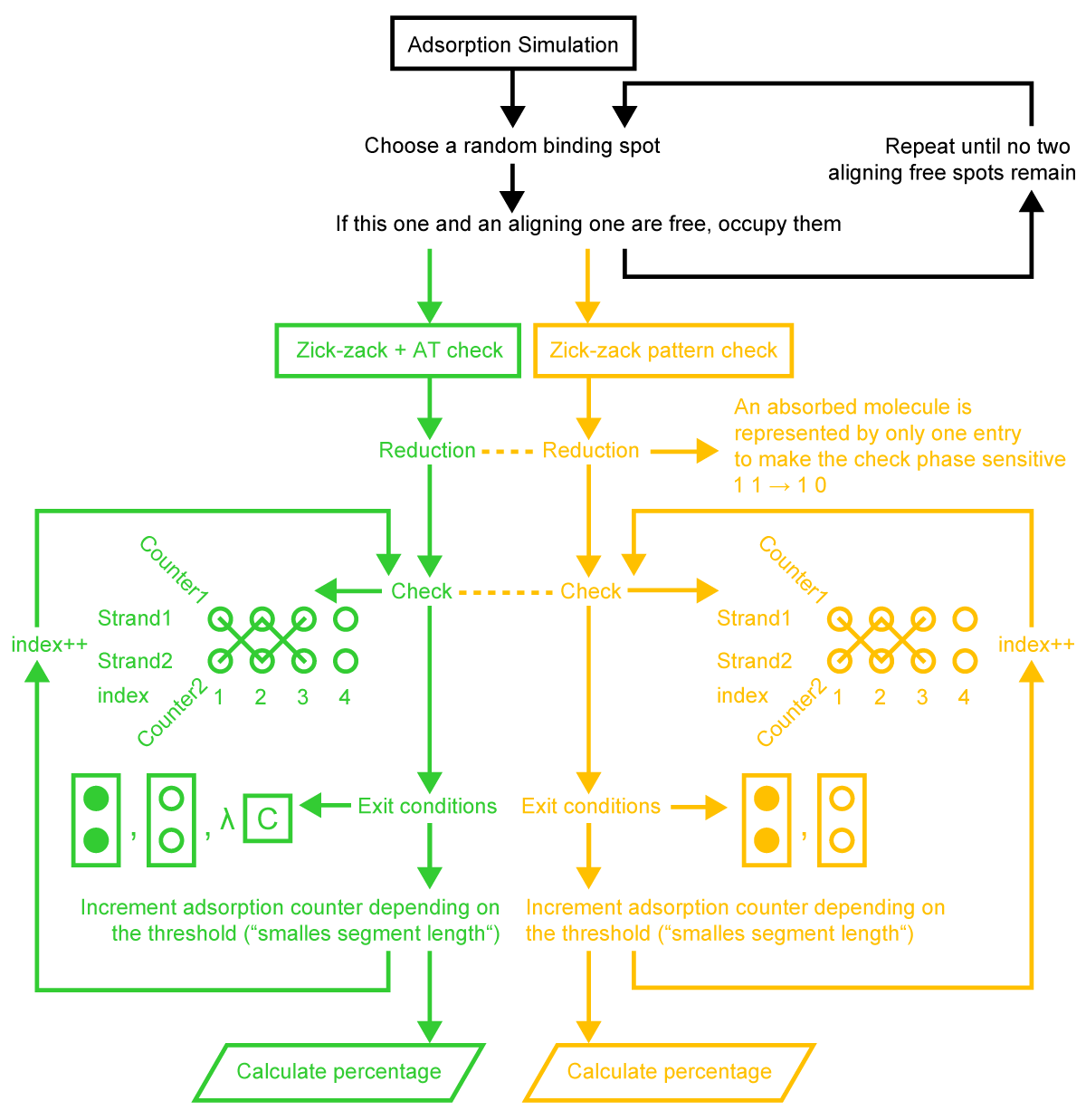


***Figure S7.*** *Pseudocode for the adsorption simulation and subsequent analysis. The adsorption routine is depicted in black. Two arrays of singular binding spots, representing the two single strands of one double helix, get subsequently and randomly filled up until no free spots remain. Since the molecules require two adjacent spots, full adsorption will leave randomly distributed singular free spots. In yellow and green depicted are the analysis routines, according to their colors in the graph of Figure 4 in the main paper. The adsorbed strand is reduced for easier identification of molecules. Then two counters are iterated over the arrays, each counting one zigzag phase. The exit conditions for the counting of one segment are that both spots of the same index are either empty or both occupied, meaning the zigzag arrangement would be interrupted either way. In addition, when a GC-pair is present (green).*

**References**

1. Jany, T.; Moreth, A.; Gruschka, C.; Sischka, A.; Spiering, A.; Dieding, M.; Wang, Y.; Samo, S. H.; Stammler, A.; Bögge, H.; Fischer von Mollard, G.; Anselmetti, D.; Glaser, T. Rational Design of a Cytotoxic Dinuclear Cu_2_ Complex That Binds by Molecular Recognition at Two Neighboring Phosphates of the DNA Backbone. *Inorg. Chem.* **2015**, *54*, 2679–2690.

2. Schwarzbich, S.; Horstmann née Gruschka, C.; Simon, J.; Siebe, L.; Moreth, A.; Wiegand, C.; Lavrentieva, A.; Scheper, T.; Stammler, A.; Bögge, H.; Fischer von Mollard, G.; Glaser, T. Stronger Cytotoxicity for Cancer Cells Than for Fast Proliferating Human Stem Cells by Rationally Designed Dinuclear Complexes. *Inorg. Chem.* **2020**, *59*, 14464–14477.

3. Drew, H. R.; Wing, R. M.; Takano, T.; Broka, C.; Tanaka, S.; Itakura, K.; Dickerson, R. E. Structure of a B-DNA Dodecamer: Conformation and Dynamics. *PNAS* **1981**, *78*, 2179–2183.

4. Bingman, C. A.; Zon, G.; Sundaralingam, M. Crystal and molecular structure of the A-DNA dodecamer d(CCGTACGTACGG). *J. Mol. Bio.* **1992**, *227*, 738–756.
